# Melatonin ameliorates cognitive memory by regulation of cAMP-response element-binding protein expression and the anti-inflammatory response in a rat model of post-traumatic stress disorder

**DOI:** 10.1186/s12868-018-0439-7

**Published:** 2018-07-04

**Authors:** Bombi Lee, Insop Shim, Hyejung Lee, Dae-Hyun Hahm

**Affiliations:** 1 Acupuncture and Meridian Science Research Center, College of Korean Medicine, Kyung Hee University, 26, Kyungheedae-ro, Dongdaemun-gu, Seoul, 02447 Republic of Korea; 2 Center for Converging Humanities, Kyung Hee University, Seoul, 02447 Republic of Korea; 3 Department of Physiology, College of Medicine, Kyung Hee University, Seoul, 02447 Republic of Korea

**Keywords:** Melatonin, Memory, Post-traumatic stress disorder, cAMP-response element-binding protein, Proinflammatory cytokines

## Abstract

**Background:**

Post-traumatic stress disorder (PTSD) is an important psychological disease that can develop following the physical experience or witnessing of traumatic events. The psychopathological response to traumatic stressors increases inflammation in the hippocampus and induces memory deficits. Melatonin (MTG) plays critical roles in circadian rhythm disorders, Alzheimer’s disease, and other neurological disorders. However, the cognitive efficiency of MTG and its mechanisms of action in the treatment of PTSD remain unclear. Thus, the present study investigated the effects of MTG on spatial cognitive impairments stimulated by single prolonged stress (SPS) in rats, an animal model of PTSD. Male rats received intraperitoneal (i.p.) administration of various doses of MTG for 21 consecutive days after the SPS procedure.

**Results:**

SPS-stimulated cognitive impairments in the object recognition task and Morris water maze were reversed by MTG treatment (25 mg/kg, i.p). Additionally, MTG significantly increased cognitive memory-related decreases in cAMP-response element-binding (CREB) protein and mRNA levels in the hippocampus. Our results also demonstrate that MTG significantly inhibited SPS-stimulated cognitive memory impairments by inhibiting the expression of proinflammatory cytokines, including tumor necrosis factor-α (TNF-α), and interleukin-6 (IL-6) in the rat brain.

**Conclusion:**

The present results indicate that MTG can be beneficial for SPS-stimulated memory impairments via changes in CREB expression and proinflammatory mediators. Thus, MTG may be a prophylactic strategy for the prevention or mitigation of the progression of some features of the PTSD pathology.

## Background

Declarative memory dysfunction is related to post-traumatic stress disorder (PTSD), which manifests following exposure to severe trauma [1]. In humans, early traumatic experiences and adversity significantly enhance one’s vulnerability to various psychiatric disorders, including memory impairment and PTSD, in adulthood [2]. The psychopathological conditions in response to traumatic stressors are caused by intrusive memories in which individuals re-experience the original traumatic experience, avoid trauma-associated events, have unpleasant recollections, avoid associated events, and exhibit negative cognition/mood, hyperarousal, and marked social impairments [3, 4]. Moreover, the persistent occurrence of extremely repulsive memory associated with the trauma and an incapacity to dissipate these fear memory are major characteristics of this disease [5]. PTSD patients also exhibit significant cognitive deficits, including damaged declarative and working memory abilities and impairments in attention and concentration [6–8]. Furthermore, cognitive impairments and memory dysfunction continually appear in conjunction with the development of PTSD [5]. The cognitive deficits observed in PTSD patients have been hypothesized to be the result of unpleasant flashback memories that temporarily interfere with the capability to procedure new memories or information [9–11]. For instance, placing a rat in a context in which it has been shocked impairs memory on an entirely different test [7, 11], such as the localization of a hidden platform in a water maze.

Single prolonged stress (SPS) is a well-validated animal model of PTSD [12]. Several studies have suggest that animals exposed to SPS exhibit states that mimic human mental disorders, including enhanced anxiety, impaired fear extinction, changes in hypothalamic-pituitary-adrenal (HPA) axis function [13], and increased cytokines in the hippocampus [14]. The hippocampus, which plays an major role in cognition and memory, is particularly vulnerability to neuronal damage caused by SPS [5, 15]. Furthermore, this damage can subsequently result in impairments in spatial learning and memory and synaptic plasticity [15, 16].

A recent study discovered that hippocampal volume is reduced in patients with PTSD [17], which emphasizes the association between stress and the loss of hippocampus neurons. In hippocampus brain-derived neurotrophic factor (BDNF) and cAMP-response element-binding protein (CREB) play major roles in pathological responses of the central nervous system (CNS) and have been related to PTSD [5, 18]. Furthermore, the hippocampus is susceptible to the inflammatory response to traumatic stress, which disrupts neuronal circuitry [19, 20], is associated with the psychosocial stress of PTSD, and alters the protein and gene expression of inflammatory mediators [21].

Currently, selective serotonin reuptake inhibitors (SSRIs) are an important mediator of the progression of PTSD and are used as potential pharmacological interventions [22, 23]. However, the used of SSRIs is contentious due to their continually reported side effects and their associated poor patient compliance [24]. Thus, there is a critical need for a novel therapeutic strategy for the treatment of PTSD [25].

Melatonin (*N*-acetyl-5-methoxytryptamine, MTG) is the major hormone released by the pineal gland at night and is secreted into the cerebrospinal fluid and circulation [26]. This neurohormone plays a important role in the modulation of the biological clock, specifically the sleep-wake cycle and the induction of physiological sleep [26, 27]. MTG plays numerous physiological roles as a regulator of circadian rhythms, a protector of mitochondria, an anti-inflammatory, an antioxidant, and a neuroprotectant agent [28–32]. The beneficial effects of MTG on neurological diseases have been widely investigated. Additionally, MTG is also thought to be involved in the modulation of complex processes, such as learning and memory [33, 34], via its binding to receptors widely distributed throughout the brain [35]. For instance, MTG in rats promotes memory in the novel object recognition task [ORT; 36, 37] and the olfactory social memory test [38]. The exogenous administration of MTG results in neuroprotective effects [39, 40] and enhance cognitive capacity [41]. MTG may exert particular therapeutic effects in patients with Alzheimer disease (AD) and Parkinson’s disease [PD; 42, 43] by protecting against neurotoxicity induced by beta-amyloid (Aβ) peptides [44, 45]. For example, MTG supplementation may weakens Aβ accumulation, inflammation, neurodegeneration, and memory impairments in AD patients and an animal model of AD [32, 41, 46]. Therefore, MTG and its receptor agonists are considered prospective therapeutic agents for AD treatment [32, 41, 46]. Additionally, some studies have shown that MTG attenuates pyramidal neuronal cell damage in the hippocampus in global cerebral ischemia [47–50]. The neuroprotective effects of MTG are related to inhibited oxidative stress and neuroinflammation [51, 52].

Based on such findings, MTG was hypothesized to alleviate SPS-stimulated memory impairment as measured by an ORT and the Morris water maze (MWM) test. Additionally, the possible mechanisms underlying the neuroprotective effects of MTG were assessed in a rat model of PTSD, and the relationships between stress-stimulated cognition and memory impairment and BDNF and CREB expressions and inflammation in the hippocampus region were evaluated. The present findings will contribute to the development of novel approaches to the treatment of trauma- and stress-related disorders, including PTSD.

## Methods

### Animals and MTG administration

Eight-week male SD rats weighing 200–230 g were obtained from Samtaco Animal Co. (Osan, South Korea). The vivarium room was kept on a 12-h light/dark cycle (lights on at 8:00, lights off at 20:00) under relative humidity of 55 ± 15% and a controlled temperature at 22 ± 2 °C. All rats were caged for 7 days to acclimatize before beginning the experimental protocol. Ethics approval was obtained from Kyung Hee University’s Institutional Animal Care and Use Committee (KHUASP(SE)-15-115). All experimental procedures were performed according to the Guide for the Care and Use of Laboratory Animals.

MTG (5, 10 and 25 mg/kg, body weight, Sigma-Aldrich Chemical Co. St. Louise, MO, USA) and fluoxetine for positive drug (10 mg/kg, FLX, fluoxetine hydrochloride; Sigma) were applied by intraperitoneally (i.p.) after the exposure to SPS for 21 days. The standard doses and period of MTG used in the this study was applied on other study [53, 54]. MTG and FLX were liquefied in 0.9% saline before use.

**Fig. 1:**
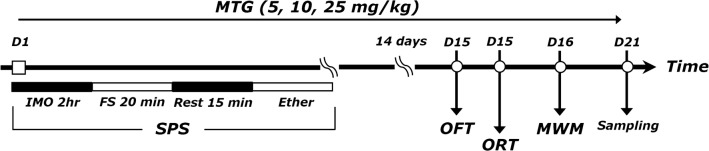
Experimental protocol for single-prolonged stress (SPS)-induced memory impairment and melatonin (MTG) treatment in rats. Groups of six or seven rats were used for each experimental condition. *OFT* open field test, *ORT* object recognition test, *MWM* Morris water maze test

The rats were randomly divided into six groups of six to seven individuals each as follows: the saline-treated group (CON group, n = 7), the SPS-stimulated plus saline-treated group (SPS group as a control, n = 7), the SPS-stimulated plus 5 mg/kg MTG-treated group (SPS+MTG5 group, n = 6), the SPS-stimulated plus 10 mg/kg MTG-treated group (SPS+MTG10 group, n = 6), the SPS-stimulated plus 20 mg/kg MTG-treated group (SPS + MTG20 group, n = 7) and the SPS-stimulated plus 10 mg/kg fluoxetine-treated group (SPS + FLX group, n = 7). The entire experimental schedules are shown in the Fig. 1.

### Single prolonged stress

Rat were exposed to SPS for 14 successive days as described by Patki’s group with a slight modification [55, 56]. Briefly, rats were restrained for 2 h on a holder and then promptly placed in a forced swimming condition for 20 min. The rats were allowed to dry and recuperate for 15 min and then exposed to ether vapor until loss of consciousness. Following the SPS procedure, rats were housed one per cage and left undisturbed for 14 days to allow PTSD-like symptoms to become apparent [55]. The other half of the rats for control group were momentarily placed in a separate area of the room during the SPS procedure. All rats were returned to the vivarium room and housed for 14 days without disturbance other.

### Object recognition task

The novel ORT was used to estimate the cognitive capability of rats. This test was essentially the same as that described by Okuda et al. [57]. Briefly, the equipment was consists square wood box and painted with black (45 × 45 × 45 cm^3^). The objects in the equipment is prepared a familiar objects and a novel object. The familiar objects (A1 and A2) to be discriminated were two similar toys as due to make objects enough heavy, so that rats could not be able to move objects. The novel object (B) was different shape and different color toy. On habituate, the rats were adapted to the object recognition box during 10 min. The test phase was started 24 h after the habituation. Rats were exposed inside the equipment with two familiar objects during 5 min. During the test phase, rats housed to the testing chamber, where rats were exposed to one novel object (B) and one of the familiar object during 5 min. The exploration (sniffing) time for the novel and familiar objects is measured. The discrimination index is calculated of discrimination between the familiar and the novel object accurate for exploration. It is expressed as: (time spent on novel object—time spent on familiar object)/(time spent on novel object + time spent on familiar object).

### Morris water maze test

After the ORT, the MWM test was used to measure the time and distance spent swimming to reach a submerged platform in the MWM test, performed as previously described [58]. MWM test was made of a spatial probe test and a place navigation test. The MWM consisted of a circular pool (200 cm diameter and 50 cm deep). The pool contained water maintained at a temperature of 22 ± 2 °C. The escape platform (15 cm diameter) in diameter was located 1.5 cm below the water in one of four sections of the pool. The hidden platform trial for acquisition test and probe trails for retention test were monitored by a video camera mounted on the ceiling, and data were analyzed by using a tracking program (S-MART: PanLab Co., Barcelona, Spain). The rats performed three training trials per day for five successively days. Each trial was terminated when the rat found the platform or after 180 s. On day 6, the platform was removed. In this probe trial, the each trial was 1 min in duration. The swimming path length, swimming speed, and time spent in the target quadrant were analyzed.

### Open field test (OFT)

Before the completion of the MWM test, the rats were exposed to the OFT. The OFT was carried out according to a previously described method [55]. In the dimly lit room, rats was exposed singly in a square black plexiglass apparatus (60 × 60 × 30 cm) and tracked by a video tracking system for 5 min. Locomotion were analyzed by the distance and speed of movements and observed by a computerized video-tracking analysis program S-MART (PanLab Co., Barcelona, Spain). The number of rearing was also manually scored by examining the records in the OFT.

### Measurement of corticosterone (CORT), BDNF, CREB and proinflammatory markers

All rats were deeply anesthetized through inhalation of isoflurane (1.2%) and were humanely sacrificed 1 day after behavioral measurement. The concentration of CORT, tumor necrosis factor-α (TNF-α), and interleukin-6 (IL-6) in the blood, and BDNF and CREB in the brain 21 days after SPS have been described previously [58]. The blood (n = 4/group) was rapidly collected via the abdominal aorta. The hippocampus (n = 4/group) was quickly removed from the rat brain in a randomized order. The CORT, TNF-α, IL-6, BDNF and CREB concentrations were measured by a competitive enzyme-linked immunosorbent assay (ELISA) using a CORT antibody (Novus Biologicals, LLC., Littleton, CO, USA), a TNF-α antibody (Abcam, Cambridge, MA, USA), an IL-6 antibody (Abcam), a BDNF antibody (R&D Systems, Minneapolis, MN, USA), and a CREB antibody (Thermo Fisher Scientific, Waltham, MA, USA) according to the manufacturer’s protocol. Detectable CORT (46–304 ng/mL), BDNF (9–45 pg/mg), CREB (1–62 pg/mg), TNF-α (4–52 pg/mL), and IL-6 (1–54 pg/mL) concentrations ranged. Intra-assay and inter assay variation ranged from 1.35–10.31% CV and 4.73–16.59% CV, respectively. 100% specificity to rat CORT, BDNF, CREB, TNF-α and IL-6 were reported in manufacturer protocol.

### Total RNA preparation and RT-PCR analysis

The expression of BDNF, CREB, TNF-α and IL-6 mRNA was evaluated by reverse transcription**-**polymerase chain reaction (RT**-**PCR) according to a previously described method [58]. In brief, total RNA was extracted from the hippocampus (n = 3/group) of each rat using TRIzol reagent (Life Technologies, Carlsbad, CA, USA) according to the manufacturer’s instructions. cDNA was synthesized from 2 µg total RNA using reverse transcriptase (Takara Bio, Otsu, Japan) with random hexamers (COSMO Genetech, Seoul, Korea), and then amplified at 57 °C for 27 cycles in the BDNF reaction, at 51 °C for 27 cycles in the CREB reaction, at 58 °C for 30 cycles in the TNF-α reaction, and at 60 °C for 30 cycles in the IL-6 reaction by PCR using Taq DNA polymerase (Takara, Kyoto, Japan) on a thermal cycler. Data were normalized against GADPH expression in the corresponding sample.

### Immunohistochemistry

The immunohistochemical analyses have been described previously [58]. The sections obtained from the brains were immunostained for CREB expression using the avidin-biotin-peroxidase complex (ABC) method. Briefly, the sections were incubated with a primary rabbit anti-CREB antibody (1:200 dilution, Cell Signaling, Boston, MA, USA) in PBS plus 0.3% Triton X-100 (PBST) for 72 h at 4 °C. Next, the sections were incubated for 120 min at room temperature with secondary antibodies (1:200 dilution, Vector Laboratories Co., Burlingame, CA, USA) in PBST containing 2% normal serum. To visualize immunoreactivity, the sections were incubated for 90 min in ABC reagent (Vectastain Elite ABC kit, Vector Labs. Co.), and then in a solution containing 3,3′-diaminobenzidine (DAB; Sigma-Aldrich) and 0.01% H_2_O_2_ for 1 min. The sections were viewed at 200× magnification, and the number of CREB-labeled cells was quantified in the hippocampus.

### Statistical analysis

All data are expressed as mean ± SEM. The data were analyzed with SPSS 13.0 (Chicago, IL, USA). Data were analyzed by the multiple way of analysis of variance (ANOVA) and Tukey’s *post hoc* tests. Between-subjects two-way ANOVA was used to analyze the effects of MTG treatment and time. In all of the analyses, differences were considered statistically significant at *p *< 0.05.

## Results

### Effect of MTG on SPS-stimulated changes in the plasma CORT level

**Fig. 2:**
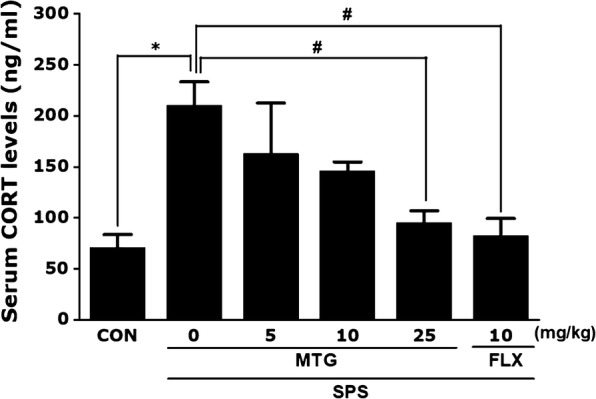
Effects of MTG on plasma corticosterone (CORT) levels in rats with SPS-induced memory impairments: assessed with an enzyme-linked immunosorbent assay (ELISA). Parameters were determined at the end of the experiments. Data are expressed as the mean ± SEM of 4 animals in each group. *p < 0.05 versus the CON group; ^#^*p *< 0.05 versus the SPS group

ELISA analysis showed that rats who underwent SPS exposure had a significantly higher plasma CORT concentration (297.87%) than rats in the saline-treated (CON) group 21 days after SPS exposure (*p *< 0.05; Fig. 2). However, administration of MTG at 25 mg/kg decreased the SPS-stimulated increase in the plasma CORT level (*p *< 0.05). However, administration of MTG at 5 or 10 mg/kg did not alter plasma CORT levels in the SPS-pretreated rats. Thus, the SPS procedure caused memory impairments in rats and was utilized to develop the PTSD or traumatic stress model in rats. The increased plasma CORT concentration in the SPS group was significantly replaced to levels similar to those in the CON group by 10 mg FLX (*p *< 0.05). This reversal display that the CORT concentration in the plasma of rats treating 25 mg/kg MTG was similar to that of rats treating 10 mg/kg FLX. Suppression of the increase in plasma CORT level by MTG also provided a base for the scientific inference that SPS-induces memory impairment in rats.

### Effects of MTG on SPS-stimulated memory impairment

**Fig. 3:**
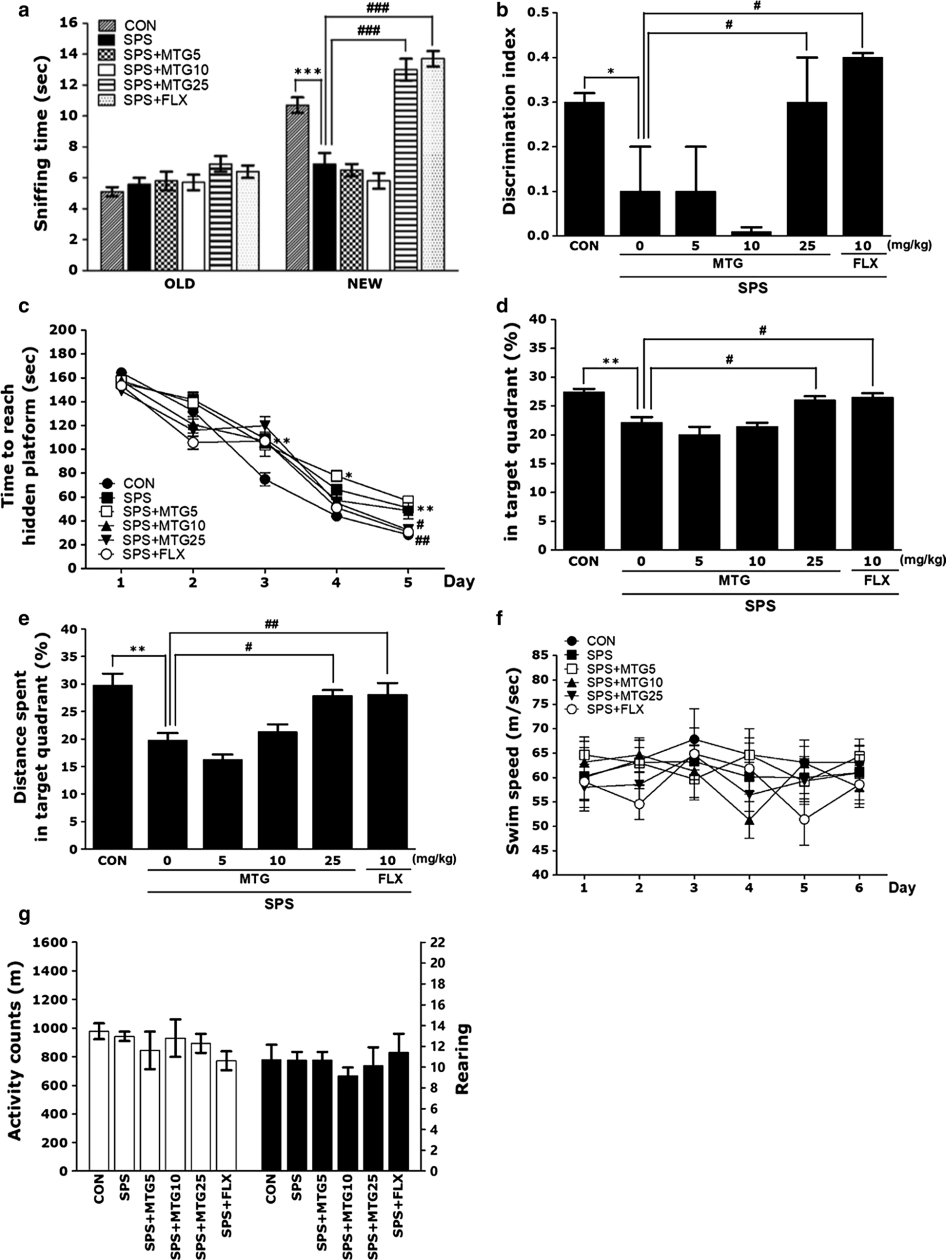
Effects of MTG on recognition memory assessed by the novel object recognition test (ORT) in which the time spent sniffing familiar and novel objects during a 3-min choice trial (**a**) and the ability to discriminate (**b**) between familiar and novel objects were measured. The Morris water maze (MWM) test was used to assess the effects of MTG on spatial learning and memory. Time to escape (latency) from the water onto a submerged platform during acquisition trials (**c**), percentages of time spent in the target quadrant (**d**), percentages of distance traversed in the target quadrant (**e**), and swimming speed (**f**) were used as outcome measures. The open field test (OFT) was used to assess the effect of MTG on locomotor activity (counts) and total number of rearing bouts (**g**). Six or seven rats were used per treatment group. Data are represented as the mean ± SEM. *p < 0.05, **p < 0.01, ***p < 0.001 versus the CON group; ^#^*p *< 0.05, ^##^*p *< 0.01, ^###^*p *< 0.001 versus the SPS group

The novel object recognition for learning and memory function was indicated by means of the exploration (sniffing) times of familiar and novel objects and by computation of the discrimination indix by the ORT (Fig. 3a and b). Analyses of sniffing times for old objects by one-way ANOVA revealed no significant differences between the groups (F(5,39) = 1.857, *p* = 0.128). There was no significant difference among the groups in sniffing time for old objects. Analyses of sniffing times for novel objects by one-way ANOVA revealed significant differences between groups (F(5,39) = 37.059, *p *< 0.001), and *post hoc* comparisons using Tukey’s test manifested a significant reduction in sniffing time for novel objects in all SPS-stimulated groups compared to the sniffing time in controls (*p *< 0.001; Fig. 3a). The negative effect of stress on recognition memory, indicated by sniffing time, was altered by MTG treatment (5 and 10 mg/kg). There were no statistically significant effects of MTG (5 and 10 mg/kg) treatment on the remaining parameters measured in the ORT. However, the rats in the SPS + MTG25 group indicated longer sniffing times for novel objects than the rats in the SPS group (*p *< 0.001). *Post*-*hoc* examination with Tukey test revealed that the discrimination index of PTSD group was significantly reduced compared to the CON group (*p *< 0.05; Fig. 3b). However, the rats in the SPS + MTG25 group indicated a higher discrimination index than the rats in the SPS group (*p *< 0.05). This difference also showed that the recovery of recognition memory after the SPS stimulated deficit was almost comparable in the SPS + MTG25 and the SPS + FLX groups.

In MWM test, SPS-stimulated rats were weak to learn during acquisition trial and retention trial. The effects of MTG treatment on swimming time to reach the submerged platform in the MWM test are shown in Fig. 3c–f. The SPS group indicated marked retardation in escape latency during all trial sessions, especially due to memory impairments resulting from SPS-stimulated learning and memory deficits.

PTSD influenced performance in the acquisition phase. More specifically, the SPS group indicated significantly enhanced latency compared with the CON group (Fig. 3c, d). ANOVA (6 × 5, treatment × time) disclosed a significant difference among groups (F(5,34) = 16.240, *p *< 0.01) and an effect of the day of training (F(4136) = 351.639, *p *< 0.01); however, a group × day interaction was not discovered (F(20,136) = 1.588, *p* = 0.064). The SPS group indicated worse performance than the CON group (*p *< 0.05 on the days 3 and 5, *p *< 0.01 on the day 4). Tukey’s *post hoc* test showed that rats in the SPS+MTG25 group had significantly reduced swimming latency compared to those in the SPS group (*p *< 0.01 on day 5). Both the 5 and 10 mg/kg MTG treatment groups still exhibited longer swimming durations than the SPS group. To examine the effect of SPS and MTG on the spatial memory of rats, performance in the probe trial on day 6 was investigated by calculating the percentages of time spent swimming in the speculated position of the platform. The swimming times and distances were reduced in the rats that swam directly to the target area where the platform had been located. The rats exposed to SPS indicated serious deficits of spatial memory performance in the MWM test (*p *< 0.01; Fig. 3d, e). A reduction in distance traveled was observed when 10 mg/kg MTG was administered to rats exposed to SPS, although this result was only marginally significant. Therefore, 10 mg/kg MTG could not completely restore the impaired memory in SPS rats. The rats in the 25 mg/kg MTG-treated group spent more time around the platform area than those in the SPS group (*p *< 0.05). Furthermore, the swimming latency in rats that received MTG was higher than the latency in the SPS group, indicating a reversal of the SPS-stimulated impairment in memory. Thus, MTG-treated rats indicated a significant improvement in the memory retention test because they spent more time in the quadrant where the platform was previously located and swam over the previous location of the platform more continually. The SPS group was not significantly different from the other groups in mean swimming speed, as analyzed by dividing the total swim distance by latency (*p* = 0.645; Fig. 3f). Based on these results, rats treated with 25 mg/kg MTG indicated greater enhancement in acquisition during the hidden platform trial and, consequently, arrived the platform more quicker than the SPS-stimulated rats. Our results also showed that the swimming latency of the SPS-stimulated rats treating 25 mg/kg MTG was similar to that of rats treating 10 mg/kg of FLX.

A parametric one-way ANOVA was executed, and as shown in Fig. 3g, no PTSD-associated differences were discovered in locomotor activity (motor function) or total number of rearings (hyperactivity) in the OFT. There was no significant difference between saline-treated rats, SPS-exposed rats, and MTG-treated rats in remarked locomotor activity (F(5,39) = 1.271, *p* = 0.322) or total number of rearings (F(5,39) = 0.337, *p* = 0.887).

Because no significant difference in locomotor activity was remarked among groups in the OFT, the remarked deficits in learning and memory in the rats exposed to SPS were not attributable to differences in locomotor activity. Rats may also indicate water-avoidance behaviors when tackled with an MWM test. However, this results demonstrate that no rats presented anxiety-like behaviors in the OFT after a stress exposure in the MWM test.

### Effects of MTG on SPS-stimulated changes in BDNF and CREB in the hippocampus

**Fig. 4:**
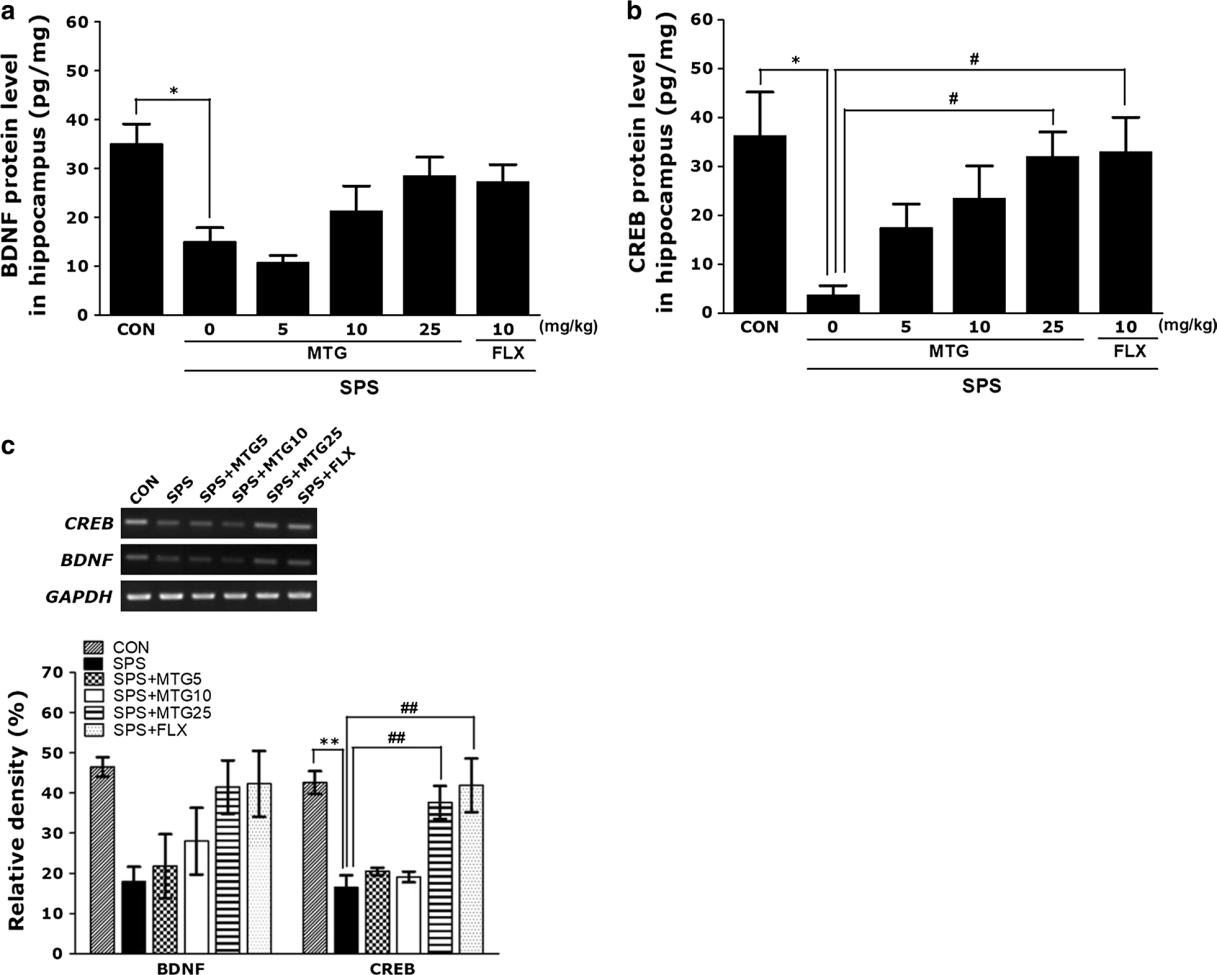
Effects of MTG on brain-derived neurotrophic factor (BDNF) and cAMP-response element-binding (CREB) protein levels (**a** and **b**) and BDNF and CREB mRNA expression in the hippocampus of rats with SPS-induced memory impairments. Polymerase chain reaction (PCR) bands on agarose gels and relative intensities (**c**). BDNF and CREB mRNA expression was normalized to the expression of glyceraldehyde 3-phosphate dehydrogenase (GAPDH) mRNA as an internal control. Parameters were determined at the end of the experiments. Data are expressed as the mean ± SEM of 4 animals in each group. *p<0.05, **p < 0.01 versus the CON group; ^#^*p *< 0.05, ^##^*p *< 0.01 versus the SPS group

Figure 4 indicates that the hippocampal levels of BDNF and CREB were significantly different among the groups. One-way ANOVA of the concentrations of BDNF and CREB in the hippocampus disclosed a significant difference among the groups [(F(5,23) = 3.961, *p *< 0.05) and ((F(5,23) = 6.133, *p *< 0.01). The *post hoc* test results showed a significant decline in BDNF and CREB concentrations in the hippocampus of the SPS group compared with those in the CON group (*p *< 0.05; Fig. 4). Furthermore, 10 mg/kg MTG could not completely reverse the decreased concentration of hippocampal BDNF and CREB observed in the SPS group. Daily administration of 25 mg/kg MTG reversed the SPS-stimulated decrease in BDNF level in the hippocampus, although this result was only marginally significant. Daily administration of 25 mg/kg MTG significantly reversed the SPS-stimulated decrease in CREB concentration in the hippocampus (*p *< 0.05). Additionally, the CREB concentration in the hippocampus of rats treating 10 mg/kg FLX was similar to that in the hippocampus of rats treating 25 mg/kg MTG.

To further investigate the effects of MTG on the expression of neurotrophic factors in the hippocampus of rats exposed to SPS, BDNF and CREB mRNA expression was analyzed by RT-PCR. Although the mRNA level of BDBF in the SPS group was lower than that in the CON group, this result was only marginally significant. However, CREB mRNA expression in the SPS group was significantly lower than that in the CON group (*p *< 0.01). The decreased expression of CREB mRNA in the SPS group was significantly reinstated to levels similar to those in the CON group by 25 mg/kg MTG (*p *< 0.01). Furthermore, CREB mRNA expression in the hippocampus of rats treating 25 mg/kg MTG was similar to that of rats treating 10 mg/kg FLX.

### Effects of MTG on SPS-stimulated changes in neuroinflammatory cytokines in the hippocampus

**Fig. 5:**
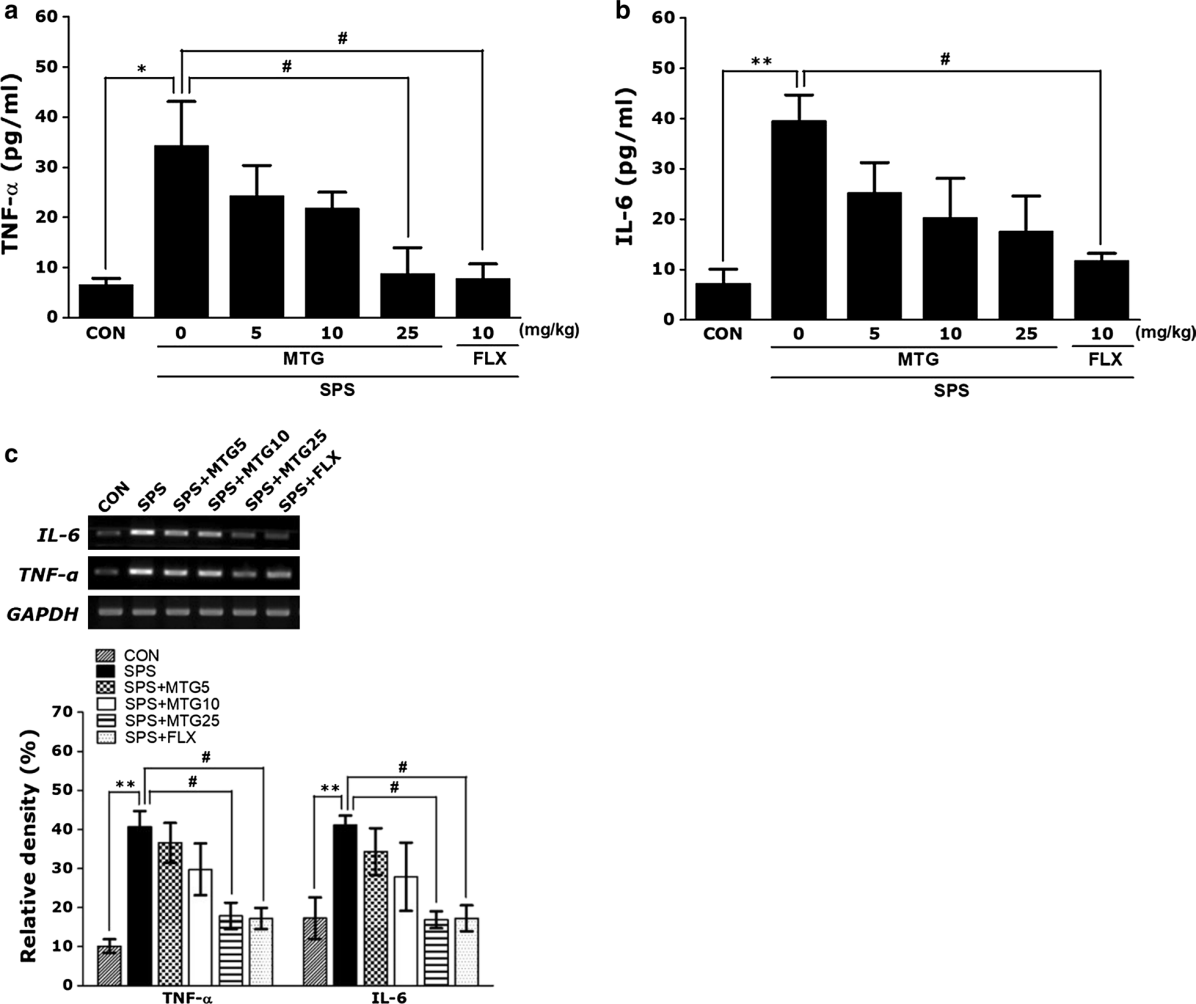
Effects of MTG on tumor necrosis factor-α (TNF-α) and interleukin-6 (IL-6) protein levels (**a** and **b**) and TNF-α and IL-6 mRNA expression in the hippocampus of rats with SPS-induced memory impairments. PCR bands on agarose gels and relative intensities (**c**). TNF-α and IL-6 mRNA levels were normalized to GAPDH levels as an internal control. Parameters were determined at the end of the experiments. Data are expressed as the mean ± SEM of 4 animals in each group. *p < 0.05, **p < 0.01 versus the CON group; ^#^*p *< 0.05 versus the SPS group

Figure 5 indicates that the hippocampal concentrations of TNF-α and IL-6 were significantly different in comparisons among the groups. One-way ANOVA of the concentrations of TNF-α and IL-6 in the hippocampus disclosed a significant difference among the groups ((F(5,23) = 4.678, *p *< 0.01) and ((F(5,23) = 4.189, *p *< 0.05). The *post hoc* test results showed a significant increase in TNF-α and IL-6 levels in the hippocampus of the SPS groups compared to those in the CON group (*p *< 0.05 and *p *< 0.01; Fig. 5). Furthermore, MTG (5 and 10 mg/kg) treatment could not completely reverse the increased concentration of hippocampal TNF-α or IL-6 observed in the SPS group. However, daily administration of 25 mg/kg MTG significantly reversed the SPS-stimulated increase in the TNF-α level in the hippocampus (*p *< 0.05), and daily treatment of 25 mg/kg MTG reversed the SPS-stimulated increase in IL-6 in the hippocampus, although this result was only marginally significant. Additionally, the TNF-α concentration in the hippocampus of rats treating 10 mg/kg FLX was similar to that of rats treating 25 mg/kg MTG.

To investigate the effects of MTG on the expression of neuroinflammatory cytokines in the hippocampus of rats exposed to SPS, the mRNA expression of TNF-α and IL-6 was analyzed by RT-PCR. The mRNA levels of TNF-α and IL-6 in the SPS group were significantly increased compared with that in the CON group (*p *< 0.01), but the increased TNF-α and IL-6 mRNA expression in the SPS group was significantly reinstated to levels similar to those in the CON group by 25 mg/kg MTG (*p *< 0.05). Finally, TNF-α and IL-6 mRNA expression in the hippocampus of rats treating 25 mg/kg MTG was similar to that of rats treating 10 mg/kg FLX.

### Effect of MTG on SPS-stimulated changes in CREB in the hippocampus

**Fig. 6:**
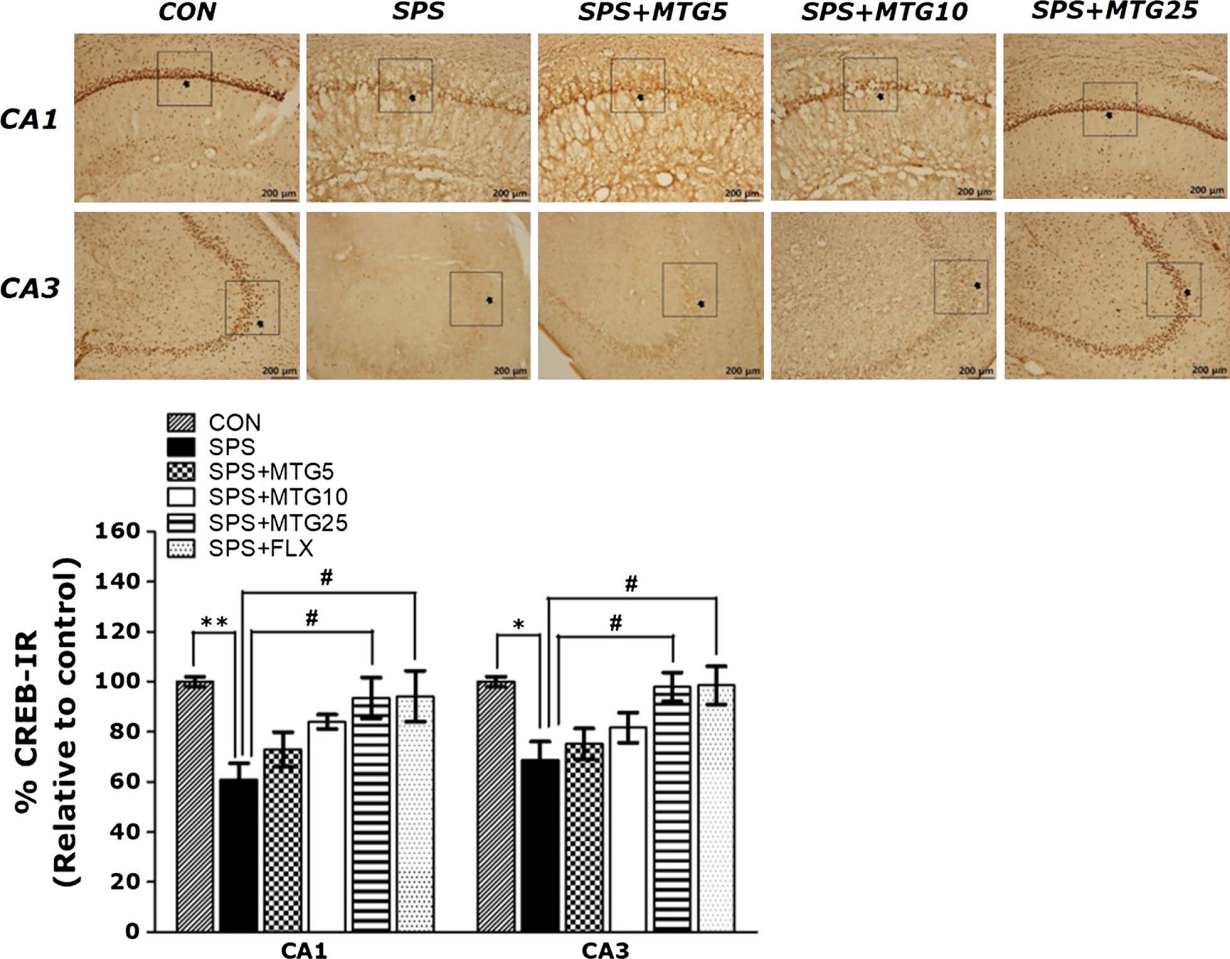
Effects of MTG on the mean number of CREB-stained hippocampal areas after the MWM test. Representative photographs and relative percentages are shown in Fig. 6. The scale bar represents 100 μm. Representative images of immunoreactive neurons are shown above each graph. Their values were calculated as a percentage of the corresponding value of the control (CON) group. Data are expressed as the mean ± SEM of 3 animals in each group. *p < 0.05, **p < 0.01 versus the CON group; ^#^*p *< 0.05 versus the SPS group

Following the behavioral tests, brain tissue from the rats were analyzed using immunohistochemistry to examine the effect of MTG treatment on the neuronal loss related to the SPS-stimulated memory deficits. The quantification of CREB immunoreactive cells in the hippocampus are shown in Fig. 6. In the SPS group, the number of CREB-immunoreactive neurons in the CA1 and CA3 of the hippocampus was decreased to 60.86 and 68.66% relative to those in the CON group, respectively. One-way ANOVA of the number of CREB immunoreactive cells disclosed a significant difference among the six groups ((5,95) = 3.868, *p *< 0.01) and ((5,95) = 3.735, *p *< 0.01). *Post hoc* comparisons showed that CREB reaction in the hippocampus of the SPS group was significantly lower than that of the CON group (*p *< 0.01 in the CA1 and *p *< 0.05 in the CA3). The number of CREB-immunoreactive neurons in the SPS + MTG25 group was significantly higher in the CA1 and CA3 of the hippocampal region than in those of the SPS group (*p *< 0.05). This results also showed that the number of CREB-activity neuronal cells in the hippocampus in rats treating 25 mg/kg MTG was similar to that in rats treating 10 mg/kg FLX.

## Discussion

The present results demonstrated that SPS-stimulated memory impairments were associated with serious impairment in performance on tests of learning and memory function as well as corresponding signs of neurodegeneration in the brain, including decreased BDNF and CREB expression and increased proinflammatory cytokine levels in the hippocampus. However, treatment with MTG in a rat model of PTSD significantly advanced cognitive functions on the ORT and enhanced the number of platform crossings in the MWM test. Additionally, MTG treatment enhanced CREB activities in the hippocampus of male rats exposed to SPS-stimulated memory impairments and inhibited the increase in proinflammatory mediators in the hippocampus of rats with SPS-stimulated PTSD symptoms.

The SPS procedure is a well-validated animal model of PTSD with high face validity that addresses the core etiological factors of this disorder, including maladaptive cognitive processes, altered neuroplasticity, and enhanced negative feedback in the HPA axis [13]. SPS in rats transiently produces several impairment in learning acquisition and short-term memory that are associated with be similar to PTSD or chronic stress [12, 13]. Therefore, in the present study, rats were subjected to SPS to mimic the psychosocial and physiological stressors associated with PTSD and then treated with various doses of MTG.

In this model, the compulsory sustaining of high CORT levels affects cognition by decreasing memory ability, which may be associated with the progression or exacerbation of traumatic stress in humans [55]. The SPS procedure increased the plasma CORT level in the rats, which is in line with chronic stress models. In rat models, compulsory maintenance of high CORT levels can affect cognition by decreasing memory ability under experimental conditions and might be closely correlated with the progression or exacerbation of a chronically stressful condition in humans [55]. The HPA axis is an important component of the neuroendocrine system that controls immune function, energy expenditure, emotions and mood, and stress [59]. HPA axis dysfunction is a specific neuroendocrine role in experimental animals and humans with PTSD [59].

MTG is the primary secretory product of the pineal gland [60, 61]. The secretion of MTG with the circadian rhythm in the blood of mammals is functionally linked to the adjustment of 24-h cycles and to circannual rhythm control. The opposing circadian alternation in MTG and HPA-related hormones may suggest a connection between these two factors. MTG has been showed to be able to reverse HPA-axis activity caused by stress, which is similar to this results [61, 62]. In addition, MTG has also been showed to cause an inhibitory effect on both stimulated and spontaneous HPA axis activation [63]. Stress stimulates the HPA axis and influences several biological effects at both the peripheral and central level. In the present study, MTG treatment after application of the SPS procedure significantly reduced serum CORT levels and improved the behavioral alternations stimulated by SPS. Our results showed that the SPS procedure produced severe impairment in the performance of cognitive functioning tests and decreases in BDNF and CREB expression in the hippocampus, suggestive of neurodegeneration in the brain. MTG restored plasma CORT to near normal levels toward the end of the 2-week treatment period, which suggests that this treatment inhibited stress-related dysfunction in the HPA axis, alleviated associated behavioral diseases, and increased CREB expression and anti-inflammatory activity. Thus, the present findings indicate that MTG treatment prevented the dysfunction of the HPA axis. These findings may elucidate the mechanisms underlying the effects of MTG in the hippocampus as well as the biochemical and behavioral signals caused by low plasma levels of CORT.

The ORT and MWM test were used to investigate the effects of MTG on cognitive memory and spatial learning and memory, respectively, and the present findings showing that SPS impaired recognition memory are consistent with those of a previous study [16]. Cognitive impairments were significantly more pronounced after exposure to SPS, as indicated by a significant enhance in time spent exploring familiar objects, reduced exploration of novel objects, and a reduction in the discrimination index. These findings suggest that, following exposure to memory-impairing agents, there is a profound deterioration in the brain that contributes to a diminished episodic memory and recognition capacity [58]. Accordingly, the this study showed that SPS significantly reduced time spent sniffing novel objects and reduced the discrimination index, whereas treatment with MTG significantly enhanced time spent sniffing novel objects and improved recognition memory. The present study used the MWM test because it is more useful for assessing spatial learning and memory in rats than other conventional mazes, such as the radial-arm maze and T-maze [58]. The MWM is a hippocampus-associated memory test that is frequently used to investigate cognitive impairment and study constant spatial learning and memory abilities and reference memory in rats [64]. In the present study, the chronically stressed animals had a significantly longer escape latency to reach the platform than the non-stressed animals and exhibited spatial learning deficits in the MWM test. The chronically stressed animals that received MTG learned faster and had shorter escape latency than the untreated chronic stress group. Moreover, compared with the non-stressed rats, the chronically stressed rats that did not receive MTG treatment exhibited poorer performances on probe trials administered 24 h after task acquisition, which is indicative of damaged memory recall and retrieval. MTG reversed these behavioral abnormalities and reinstated spatial learning and memory in the chronically stressed rats. A similar effect was seen following chronic treatment with fluoxetine [65]. Thus, the this findings support and prove the hypothesis that MTG ameliorates spatial learning and memory impairment caused by traumatic stress.

An OFT was performed to rule out the potentially confounding effects of motor deficits, which could influence the outcomes of behavioral tests of anxiety and depression. However, no significant individual differences in locomotor activity were discovered between the groups, which suggests that MTG treatment did not affect sensorimotor performance. Thus, the improved performance in the MWM test was more likely due to improved learning and memory than to differences in limb flexibility, motor output, or sensorimotor function. Furthermore, no rats in either group appeared anxiety-like behaviors in the OFT after stress exposure in the MWM test, which indicates that MTG did not alter psychomotor function or active responses as measured by performance in the MWM test.

To identify additional MTG-related mechanisms underlying the improvements in memory, the effects of MTG on BDNF and CREB levels in the hippocampus were investigated. The change in the levels of BDNF and CREB proteins in the brain supplies a novel treatment strategy for the amelioration of memory impairment [66]. In addition to its roles in neuronal cell survival and the prevention of neurodegeneration, recent experimental evidence strongly supports the role of BDNF in the regulation of synaptic function and plasticity in the CNS for learning and memory processes [67]. However, in the present study, no significant individual differences in BDNF level were observed following treatment with MTG, which suggests that MTG did not affect the BDNF level. CREB is also believed to play a critical role in the formation of memories [66]. SPS-stimulated memory impairment are associated with significant reductions in CREB mRNA expression in the hippocampus as well as poor performance on hippocampus-dependent tests [5, 65]. In the present study, MTG treatment significantly reversed SPS-stimulated decreases in CREB mRNA expression, which suggests that the beneficial effects of MTG were mediated by increases in CREB expression that may be associated with enhanced neuronal function and performance in learning and memory tests. Furthermore, the present findings indicate that there is a correlation between protein and gene function and decreased CREB expression in the hippocampus.

Additionally, the current results also strongly suggest a close correlation between hippocampal CREB expression and number of CREB-immunoreactive neurons in the hippocampus. CREB dysfunction interrupts hippocampus-dependent memory formation, and CREB has been suggested to be required for memory solidity [68, 69]. Thus, BDNF transcriptional activity, up-regulated by CREB, may also play an important role in adaptive neuronal activations underlying memory function [70, 71]. The administration of MTG is proposed to significantly prevent the reduction in CREB in the hippocampus stimulated by SPS exposure, leading to memory deficits. Although MTG reversed the decrease in CREB expression in the hippocampus to some degree, the effect of MTG on other upstream or downstream pathways involving CREB was not determined [72]. The changes in CREB associated with memory impairment have been found to be accompanied by enhances in the phosphorylation of extracellular signal-related kinase [72]. Thus, further studies will be necessary to clarify more precisely the effects of MTG on the CREB-mediated signaling pathway.

In the present study, SPS also significantly increased the expression of TNF-α and IL-6 in the hippocampus, which ultimately led to a chronic neuroinflammatory activation in the brain. Many studies have demonstrated that SPS-stimulated TNF-α and IL-1β expression are upregulated in PTSD and that these cytokines play a role in several events associated with the pathological cascade of PTSD [73]. Thus, inflammatory reactions may be associated with the pathogenesis of degenerative changes as well as cognitive impairments [74]. In this study, SPS induced an increase in the levels of the proinflammatory cytokines TNF-α and IL-6 and produced learning and memory impairment. However, MTG inhibited the increased expression of TNF-α and IL-6 in SPS-treated rats. MTG decreased the SPS-stimulated increase in the mRNA expression of TNF-α and IL-6, eventually resulting in the reversal of chronic inflammation and the amelioration of persistent brain dysfunction [65, 73]. According to the inflammation hypothesis, memory impairments in PTSD are due to selective and irreversible dysfunction and chronic inflammation in the brain [65, 73]. Thus, the anti-inflammatory effects of MTG are proposed here to significantly reverse the impaired memory retention and the increased expression of proinflammatory cytokines.

## Conclusions

In summary, the present study demonstrated that SPS impaired neuronal function and produced associated memory and cognitive impairment in a rat model of progressive memory impairment in neurodegenerative disease. This was evidenced by performance on the ORT and MWM tests and by protein and gene expression analyses of CREB and BDNF. However, MTG treatment significantly attenuated the SPS-stimulated deficits as indicated by improved cognitive function on the behavioral tests, increased CREB expression, and normalization of the HPA axis. Moreover, MTG suppressed increases in the mRNA expressions of TNF-α and IL-6, which are proinflammatory mediators, in the hippocampus. Thus, MTG may be a useful agent for the prevention of neuronal impairments and the attenuation of anti-inflammatory effects such as those observed in patients with PTSD.

## Abbreviations

PTSD: Post-traumatic stress disorder
MTG: Melatonin
SPS: Single prolonged stress
CREB: cAMP-response element-binding protein
HPA: Hypothalamic-pituitary-adrenal
BDNF: Brain-derived neurotrophic factor
SSRIs: Selective reuptake inhibitors
AD: Alzheimer disease
PD: Parkinson’s disease
ORT: Object recognition task
MWM: Morris water maze
SD: Sprague-Dawley
FLX: Fluoxetine
CORT: Corticosterone
OFT: Open field test
TNF-α: Tumor necrosis factor-α
IL-6: Interleukin-6
ELISA: Enzyme-linked immunoassay
RT-PCR: Reverse transcription**-**polymerase chain reaction
GAPDH: Glyceraldehyde-3-phosphate dehydrogenase
PBS: Phosphate-buffered saline
ABC: Avidin-biotin-peroxidase complex
DAB: 3,3′-Diaminobenzidine
ANOVA: Analysis of variance
